# RETRACTED: Khalil-ur-Rehman et al. Impact of Substantive Staging and Communicative Staging of Sustainable Servicescape on Behavioral Intentions of Hotel Customers through Overall Perceived Image: A Case of Boutique Hotels. *Int. J. Environ. Res. Public Health* 2021, *18*, 9123

**DOI:** 10.3390/ijerph23040533

**Published:** 2026-04-20

**Authors:** Khalil-ur-Rehman, Mohammad Adnan, Naveed Ahmad, Miklas Scholz, Muhammad Khalique, Rana Tahir Naveed, Heesup Han

**Affiliations:** 1 Leads Business School, Lahore Leads University, Lahore 54000, Pakistan; khalil@leads.edu.pk; 2 Swiss Business School (SBS), 8302 Kloten, Switzerland; adnan.abdulhamid@gmail.com; 3 Faculty of Management Studies, University of Central Punjab, Lahore 54000, Pakistan; naveed.ahmad@vu.edu.pk; 4 Department of Management Sciences, Virtual University of Pakistan, Lahore 54000, Pakistan; 5 Division of Water Resources Engineering, Department of Building and Environmental Technology, Faculty of Engineering, Lund University, P.O. Box 118, 221 00 Lund, Sweden; 6 Department of Civil Engineering Science, School of Civil Engineering and the Built Environment, University of Johannesburg, Kingsway Campus, PO Box 524, Aukland Park, Johannesburg 2006, South Africa; 7 Department of Town Planning, Engineering Networks and Systems, South Ural State University (National Research University), 76, Lenin Prospekt, Chelyabinsk 454080, Russia; 8 Institute of Environmental Engineering, Wroclaw University of Environmental and Life Sciences, ul. Norwida 25, 50-375 Wrocław, Poland; 9 Faculty of MUST Business School, Mirpur University of Science and Technology (MUST), Mirpur 10250, Pakistan; drmkhalique.dbms@must.edu.pk; 10 Faculty of Entrepreneurship and Business, Universiti Malaysia Kelantan (UMK), Taman Bendahara, Pengkalan Chepa 16100, Malaysia; 11 Department of Economics and Business Administration, Division of Management and Administrative Sciences, University of Education, Lahore 54000, Pakistan; tahir.naveed@ue.edu.pk; 12 College of Hospitality and Tourism Management, Sejong University, 98 Gunja-Dong, Gwanjin-Gu, Seoul 143-747, Korea

The journal retracts the article titled “Impact of Substantive Staging and Communicative Staging of Sustainable Servicescape on Behavioral Intentions of Hotel Customers through Overall Perceived Image: A Case of Boutique Hotels” [1], cited above.

Following publication, the Editorial Office identified concerns regarding the article’s alignment with the journal’s scope, as well as unintentional discrepancies identified in the authorship attribution [1].

In accordance with the journal’s standard procedures, the Editorial Office and Editorial Board conducted a review of the editorial process and confirmed that the subject matter of this article did not adequately align with the intended scope of this journal. Furthermore, despite authors’ cooperation, certain administrative and procedural requirements regarding the manuscript's documentation could not be fully reconciled with the journal’s latest verification protocols. Consequently, the Editorial Board has decided to retract this publication, as per MDPI’s retraction policy.

This retraction was approved by the Editor-in-Chief of the journal *International Journal of Environmental Research and Public Health*.

The authors did not agree to this retraction.
